# Sagittal Abdominal Diameter Is an Independent Predictor of All-Cause and Cardiovascular Mortality in Incident Peritoneal Dialysis Patients

**DOI:** 10.1371/journal.pone.0077082

**Published:** 2013-10-22

**Authors:** Mi Jung Lee, Dong Ho Shin, Seung Jun Kim, Dong Eun Yoo, Kwang Il Ko, Hyang Mo Koo, Chan Ho Kim, Fa Mee Doh, Hyung Jung Oh, Jung Tak Park, Seung Hyeok Han, Tae-Hyun Yoo, Kyu Hun Choi, Shin-Wook Kang

**Affiliations:** 1 Department of Internal Medicine, College of Medicine, Yonsei University, Seoul, Korea; 2 Severance Biomedical Science Institute, Brain Korea 21, Yonsei University, Seoul, Korea; University of Louisville, United States of America

## Abstract

**Backgrounds and Aims:**

Visceral fat has a crucial role in the development and progression of cardiovascular disease, the major cause of death in end-stage renal disease (ESRD). Although sagittal abdominal diameter (SAD), as an index of visceral fat, significantly correlated with mortality in the general population, the impact of SAD on clinical outcomes has never been explored in ESRD patients. Therefore, we sought to elucidate the prognostic value of SAD in incident peritoneal dialysis (PD) patients.

**Methods:**

We prospectively determined SAD by lateral abdominal X-ray at PD initiation, and evaluated the association of SAD with all-cause and cardiovascular mortality in 418 incident PD patients.

**Results:**

The mean SAD was 24.5±4.3 cm, and during a mean follow-up of 39.4 months, 97 patients (23.2%) died, and 49.4% of them died due to cardiovascular disease. SAD was a significant independent predictor of all-cause [3rd versus 1st tertile, HR (hazard ratio): 3.333, 95% CI (confidence interval): 1.514–7.388, P = 0.01; per 1 cm increase, HR: 1.071, 95% CI: 1.005–1.141, P = 0.03] and cardiovascular mortality (3rd versus 1st tertile, HR: 8.021, 95% CI: 1.994–32.273, P = 0.01; per 1 cm increase, HR: 1.106, 95% CI: 1.007–1.214, P = 0.03). Multivariate fractional polynomial analysis also showed that all-cause and cardiovascular mortality risk increased steadily with higher SAD values. In addition, SAD provided higher predictive value for all-cause (AUC: 0.691 vs. 0.547, P<0.001) and cardiovascular mortality (AUC: 0.644 vs. 0.483, P<0.001) than body mass index (BMI). Subgroup analysis revealed higher SAD (≥24.2 cm) was significantly associated with all-cause mortality in men, women, younger patients (<65 years), and patients with lower BMI (<22.3 kg/m^2^).

**Conclusions:**

SAD determined by lateral abdominal X-ray at PD initiation was a significant independent predictor of all-cause and cardiovascular mortality in incident PD patients. Estimating visceral fat by SAD could be useful to stratify mortality risk in these patients.

## Introduction

Cardiovascular disease is the most common cause of death in end-stage renal disease (ESRD) patients [1]. In addition to advanced age, hypertension, diabetes, smoking, and dyslipidemia, obesity has also been established as a risk factor for cardiovascular morbidity and mortality in the general population [2]–[5]. Contrary to the general population, some previous epidemiologic studies have shown that high body mass index (BMI) is associated with improved outcome in ESRD patients on hemodialysis (HD), which is referred to as “reverse epidemiology” [6], [7]. In patients with peritoneal dialysis (PD), however, the association between BMI and mortality has not been consistent [8]–[10]. Snyder et al [9] demonstrated a significant survival advantage in PD patients with high BMI, while a cohort study by McDonald et al [8] revealed that obesity at PD initiation was an independent risk factor for death and technique failure. Meanwhile, the United States Renal Data System Dialysis Morbidity and Mortality Wave II Study failed to find any survival advantage associated with high BMI in PD patients [10].

To date, accumulating evidence has shown that central obesity predicts clinical outcomes better than total obesity in the general population and in patients with various diseases, including chronic kidney disease (CKD) [11]–[17]. Waist-to-hip ratio (WHR) was demonstrated to be a better predictor of cardiovascular risk than BMI in patients with moderate chronic kidney disease [13]. In addition, waist circumference (WC) as well as WHR presented stronger prognostic value for all-cause and cardiovascular mortality than BMI in ESRD patients [15]. On the other hand, among the components of central obesity, visceral fat is more metabolically active than subcutaneous fat, and a number of studies have found that visceral fat is significantly associated with inflammation, insulin resistance, and atherosclerosis [18]–[21]. Considering the high mortality rates derived from cardiovascular disease in ESRD patients and an insufficient power of WC, WHR, and abdominal conicity index to discriminate between visceral and subcutaneous fat, the exploration of a simple and reliable anthropometric index representing visceral fat has clinical relevance in these patients.

Sagittal abdominal diameter (SAD), which measures the anteroposterior diameter of the abdomen, significantly correlated with visceral fat measured by computed tomography (CT), magnetic resonance imaging (MRI), or dual-energy X-ray absorptiometry in previous studies [22]–[25]. In addition, SAD was a more powerful anthropometric index associated with cardio-metabolic risk factors and insulin resistance than BMI, WC, and WHR [26]–[28]. Furthermore, several large cohort studies have demonstrated the prognostic value of SAD in the general population [29]–[31]. However, the impact of SAD on clinical outcome has never been explored in ESRD patients. Therefore, we investigated whether SAD on lateral abdominal X-ray at PD initiation was an independent predictor of mortality in incident PD patients.

## Methods

### Ethics statement

The study was carried out in accordance with the Declaration of Helsinki and approved by the Institutional Review Board of Yonsei University Health System (YUHS) Clinical Trial Center. We obtained informed written consent from all participants involved in the present study.

### Subjects

All consecutive ESRD patients over 18 years of age who started PD at YUHS between January 2005 and July 2010 were initially screened for enrollment in this prospective observational study. Among a total of 533 incident PD patients, we excluded 21 patients who had history of malignancy (n = 14), active infection (n = 4), or decompensated liver disease (n = 3) at the time of enrollment. We also excluded 94 patients who discontinued PD within the first 90 days after PD initiation due to following reasons: loss to follow-up (n = 39), death (n = 37), transfer to HD (n = 12), transplantation (n = 4), and recovery of kidney function (n = 2). Therefore, remaining 418 patients were included in the final analysis.

### Clinical and Biochemical Data Collection

A well-trained examiner used a questionnaire to collect demographic data. Traditional cardiovascular risk factors such as age, hypertension, diabetes mellitus, smoking history, previous history of cardiovascular disease, and PD modality were recorded. Smoking status was classified as current, former, or never. Cardiovascular disease was defined as a history of coronary, cerebrovascular, or peripheral vascular disease: coronary disease was defined as a history of angioplasty, coronary artery bypass grafts, myocardial infarction, or angina and cerebrovascular disease as a history of transient ischemic attack, stroke, or carotid endarterectomy, while peripheral vascular disease was defined as a history of claudication, ischemic limb loss and/or ulceration, or peripheral revascularization procedure. BMI and biochemical data were measured at the time of study enrollment. Patients were weighed in light clothing, and height was measured with no shoes. BMI was calculated as weight/height^2^ (kg/m^2^). Blood was taken after a 12-hour overnight fast, and the following laboratory data were measured: hemoglobin, blood urea nitrogen, creatinine, albumin, triglyceride, total cholesterol, low-density lipoprotein cholesterol, high-density lipoprotein cholesterol, calcium (Ca), phosphorous (P), and intact parathyroid hormone (iPTH) concentrations. In addition, high-sensitivity C-reactive protein (hs-CRP) levels were determined by a latex-enhanced immunonephelometric method using a BNII analyzer (Dade Behring, Newark, DE, USA). Residual renal function was estimated by a 24-hour urine collection. To reflect the actual situation, usual overnight dialysate volume and glucose concentrations were not changed for this study. Kt/V urea was determined from the total loss of urea nitrogen in spent dialysate using PD Adequest 2.0 for Windows software (Baxter Healthcare, Deerfield, IL, USA).

### Echocardiography

Echocardiography was performed on a day close to the time of discharge based on the imaging protocol recommended by the American Society of Echocardiography [32], to assess the cardiac function of the patients. Left ventricular (LV) systolic function was defined by LV ejection fraction using a modified biplane Simpson’s method from the apical two-chamber and four-chamber views.

### Assessment of SAD by Lateral Abdominal X-ray

Lateral abdominal X-rays were taken in all subjects in a supine position with the knee bent after dialysate were removed. SAD was defined as the anterior-posterior distance, from skin to skin, at the L4-L5 intervertebral disc level on a lateral abdominal X-ray. Two trained medical doctors blinded to the patients’ clinical data reviewed the X-rays and determined the SAD using an electronical measure on a digital radiologic workstation (Centricity Enterprise Web V3.0; GE Healthcare, Chalfont, UK). Any discrepancies were resolved by an independent third observer.

### Follow-up and Endpoints

Participants were followed up at intervals of 3 months through October 31, 2011. All deaths and hospitalizations were recorded in the serious adverse event database. Mortality events were retrieved from the database and carefully reviewed to determine all-cause and cardiovascular mortality. Cardiovascular mortality was defined as death from myocardial infarction or ischemia, congestive heart failure, pulmonary edema, cerebral infarction, or peripheral vascular disease. When a patient died within 60 days after transfer to HD, the death was regarded as a mortality event. Loss to follow-up, renal transplantation, transfer to HD, or recovery of renal function after the first 90 days of PD initiation were censored at the end of PD treatment.

### Statistical Analysis

Continuous variables were expressed as mean ± SD, and categorical variables were expressed as a number (percentage). Since hs-CRP was not normally distributed, log-transformed values were used. Subjects were divided into three groups according to the SAD tertiles. To compare the baseline characteristics according to the SAD tertiles, ANOVA and chi-square tests were used for continuous variables and categorical variables, respectively. Pearson’s correlation analysis was performed to estimate the association between SAD and other continuous variables. Cumulative survival curves were generated by the Kaplan-Meier method, and between-group survival was compared by a log-rank test. Independent prognostic values of SAD for all-cause and cardiovascular mortality were ascertained by Cox proportional hazards regression models, which included traditional risk factors (age, sex, presence of diabetes and previous cardiovascular disease, smoking status, systolic blood pressure, and the use of lipid-lowering therapy), BMI, and biochemical data associated with inflammation and nutrition in ESRD patients (hemoglobin, albumin, Ca×P products, hs-CRP concentrations). The prognostic value of SAD was also evaluated in a fractional polynomial model. In addtion, the predictive values of SAD and BMI for all-cause and cardiovascular mortality were compared using receiver operating characteristic (ROC) curve analysis with the calculated area under the ROC curve (AUC). Further subgroup analysis was performed to elucidate the association of SAD with mortality according to age, sex, and BMI. Age was classified as two groups (<65 or ≥65 years), and SAD and BMI were dichotomized by the median values (SAD, <24.2 cm or ≥24.2 cm; BMI, <22.3 kg/m^2^ or ≥22.3 kg/m^2^). Statistical analysis was performed using SPSS for Windows version 18.0 (SPSS Inc., Chicago, IL, USA) and STATA (version 11.0, StataCorp, www.stata.com). A *P* value less than 0.05 was considered statistically significant.

## Results

### Clinical Characteristics and Echocardiographic Findings According to the SAD Tertiles

The baseline characteristics of the subjects according to the SAD tertiles are shown in Table 1. The mean age was 55.9±13.7 years (21–80 years), and 237 patients (56.6%) were male. Overall, the mean SAD was 24.5±4.3 cm, and the mean SADs for each tertile were 19.6±1.7, 24.4±1.2, and 29.5±1.9 cm. Diabetic nephropathy was the most common cause of ESRD in all tertiels, followed by chronic glomerulonephritis. The mean age, proportion of males, proportion of patients with diabetes and previous history of cardiovascular disease, and proportion of patients taking lipid-lowering agents increased from the lowest to highest SAD tertile. In addition, BMI and hs-CRP levels were significantly higher, while albumin concentrations were significantly lower in the highest SAD tertile group. On the other hand, there were no significant differences in smoking status, PD modality, weekly Kt/V urea, residual renal function, systolic blood pressure, hemoglobin, serum glucose, creatinine, the ratio of total cholesterol to high-density lipoprotein cholesterol, Ca×P products, the use of antihypertensive drugs and phosphate binders, and LV ejection fraction among the three groups.

**Table 1:** Baseline characteristics of the subjects according to the SAD tertiles.

		Tertiles of SAD			
		(minimun-maximum, cm)			
		Tertile 1	Tertile 2	Tertile 3	
	All	(14.1–22.2)	(22.3–26.9)	(27.0–35.9)	
Characteristics	(*n* = 418)	(*n* = 139)	(*n* = 139)	(*n* = 140)	*P*
SAD (cm)	24.5±4.3	19.6±1.7	24.4±1.2	29.5±1.9	<0.001
Age (years)	55.9±13.7	49.6±13.6	57.7±13.2	60.2±12.2	<0.001
Male, *n* (%)	237 (56.6%)	62 (44.6%)	87 (62.5%)	88 (62.9%)	<0.001
Diabetes mellitus, *n* (%)	196 (46.8%)	47 (33.8%)	69 (49.6%)	80 (57.1%)	<0.001
Cardiovascular disease, *n* (%)	145 (34.6%)	30 (21.5%)	52 (37.4%)	63 (45.0%)	<0.001
Smoking status, *n* (%)					0.12
Current	7 (1.7%)	1 (0.7%)	3 (2.2%)	3 (2.1%)	
Former	130 (31.1%)	33 (23.7%)	46 (33.1%)	51 (36.4%)	
Never	281 (67.2%)	105 (75.5%)	90 (64.7%)	86 (61.4%)	
Primary renal disease, *n* (%)					0.62
Diabetic nephropathy	170 (40.6%)	46 (33.0%)	62 (44.6%)	62 (44.2%)	
Glomerulonephritis	114 (27.2%)	39 (28.0%)	37 (26.6%)	38 (27.1%)	
Hypertensive nephrosclerosis	33 (7.8%)	12 (8.6%)	10 (7.1%)	11 (7.8%)	
Polycystic kidney disease	5 (1.1%)	2 (1.4%)	2 (1.4%)	1 (0.7%)	
Others/Unknown	96 (22.9%)	40 (28.7%)	28 (20.1%)	28 (20.0%)	
PD modality, *n* (%)					0.79
CAPD	385 (92.1%)	129 (92.8%)	127 (91.4%)	129 (92.1%)	
APD	33 (7.9%)	10 (7.2%)	12 (8.6%)	11 (7.9%)	
Kt/V urea (per week)	2.2±0.6	2.1±0.5	2.2±0.6	2.3±0.6	0.16
RRF (mL/min/1.73 m^2^)	6.6±3.6	6.5±4.1	6.9±3.2	6.3±3.7	0.29
Systolic blood pressure (mmHg)	139.8±20.3	139.6±19.8	140.0±20.3	139.7±20.8	0.9
BMI (kg/m^2^)	22.6±3.1	20.4±2.0	22.4±2.2	25.1±3.1	<0.001
Hemoglobin (g/L)	92±15	93±15	91±15	91±15	0.36
Glucose (mmol/L)	6.4±2.9	6.2±2.5	6.6±3.3	6.5±2.8	0.45
Blood urea nitrogen (mmol/L)	2.4±1.0	2.6±1.0	2.5±0.9	2.4±1.0	0.59
Creatinine (µmol/L)	681±274	654±265	663±274	619±274	0.07
Albumin (g/L)	35±6	36±6	35±6	34±5	0.01
Triglyceride (mmol/L)	1.3±0.6	1.3±0.5	1.2±0.6	1.3±0.6	0.38
Total cholesterol (mmol/L)	3.8±1.1	4.1±1.1	4.0±1.2	3.9±1.0	0.41
LDL-C (mmol/L)	2.7±1.2	3.0±1.7	2.5±1.0	2.5±1.1	0.28
HDL-C (mmol/L)	0.9±0.2	1.0±0.2	0.9±0.1	0.8±0.2	0.67
Total-to-HDL-C ratio	4.7±2.0	5.0±3.0	4.3±1.7	4.8±1.0	0.78
Ca×P product (mmoL^2^/L^2^)	3.4±1.0	3.4±1.0	3.5±0.9	3.4±1.0	0.61
iPTH (ng/L)	195.7±168.3	207.5±159.2	176.1±166.4	203.7±179.6	0.32
Log hs-CRP (mg/L)	−0.1±0.8	−0.3±0.9	−0.1±0.8	0.1±0.7	<0.001
Lipid-lowering agents, *n* (%)	145 (34.6%)	38 (27.3%)	50 (36.0%)	57 (40.7%)	0.01
Antihypertensive drugs, *n* (%)					
RAS blockers	320 (76.5%)	104 (74.8%)	106 (76.2%)	110 (78.5%)	0.34
Beta-blockers	216 (51.6%)	68 (48.9%)	69 (49.6%)	79 (56.4%)	0.11
Calcium channel blockers	257 (61.4%)	83 (59.7%)	84 (60.4%)	90 (64.3%)	0.64
Phosphate binders, *n* (%)					0.81
Calcium-based	215 (51.4%)	71 (51.0%)	70 (50.3%)	74 (52.8%)	
Non calcium-based	32 (7.6%)	10 (7.1%)	10 (7.1%)	12 (8.5%)	
LV ejection fraction (%)	56.0±13.6	53.8±11.3	56.5±11.6	56.7±17.4	0.81

*Note:* Data are expressed as mean ± standard deviation or number of patients (percent).

*Abbreviations:* SAD, sagittal abdominal diameter; PD, peritoneal dialysis; CAPD, continuous ambulatory peritoneal dialysis; APD, automated peritoneal dialysis; Kt/V, fractional urea clearance; RRF, residual renal function; BMI, body mass index; LDL-C, low-density lipoproein cholesterol; HDL-C, high-density lipoprotein cholesterol; Ca, calcium; P, phosphorous; iPTH, intact parathyroid hormone; hs-CRP, high-sensitivity C-reactive protein; RAS, Renin-angiotensin system; LV, left ventricular.

### Association of SAD with Clinical and Biochemical Parameters

In the whole subjects, SAD was positively correlated with age (*r* = 0.35, *P*<0.001), BMI (*r* = 0.64, *P*<0.001), and serum hs-CRP concentrations (*r* = 0.28, *P*<0.001), whereas there was an inverse correlation between SAD and serum albumin levels (*r* = −0.14, *P*<0.001). Even when males and females were analyzed separately, the associations of SAD with age, BMI, and serum albumin and hs-CRP concentrations remained significant (Table 2).

**Table 2:** Pearson’s correlation test for the association of SAD with clinical and biochemical variables.

Characteristics	All	Men	Women
	(*n = *481)	(*n* = 241)	(*n* = 177)
Age	0.35*	0.31*	0.41*
Kt/V urea (per week)	0.14	0.13	0.15
Systolic blood pressure	−0.02	0.01	0.09
BMI	0.64*	0.59*	0.68*
Hemoglobin	−0.06	−0.06	−0.08
Glucose	0.08	0.07	0.08
Blood urea nitrogen	−0.07	−0.01	0.16
Creatinine	−0.21	−0.17	−0.31
Albumin	−0.14*	−0.16*	−0.10*
Total cholesterol	−0.06	−0.09	0.02
LDL-C (mmol/L)	−0.11	−0.29	0.01
HDL-C (mmol/L)	−0.13	−0.15	−0.11
Total-to-HDL-C ratio	0.11	0.15	0.10
Ca×P product	−0.05	0.11	0.01
iPTH	−0.01	−0.01	0.01
Log hs-CRP	0.28*	0.32*	0.25*

*Abbreviations:* SAD, sagittal abdominal diameter; Kt/V, fractional urea clearance; BMI, body mass index; LDL-C, low-density lipoproein cholesterol; HDL-C, high-density lipoprotein cholesterol; Ca, calcium; P, phosphorous; iPTH, intact parathyroid hormone; hs-CRP, high-sensitivity C-reactive protein.

* *P*<0.001.

### SAD as an Independent Risk Factor for All-cause and Cardiovascular Mortality

During a mean follow-up duration of 39.4±21.3 months, 97 patients (23.2%) died, and 49.4% of them died due to cardiovascular disease. All-cause and cardiovascular mortality rates were significantly and substantially increased with increasing SAD tertiles. In the highest SAD tertile, all-cause and cardiovascular death rates were 14.5 and 7.1 per 100 Person-Years, respectively, which were significantly higher than those of the lowest (2.5 and 0.7 per 100 Person-Years, respectively, *P*<0.001) and middle SAD tertile (8.5 and 4.9 per 100 Person-Years, respectively, *P*<0.001) (Table 3).

**Table 3:** All-cause and cardiovascular death rates according to the SAD tertiles.

Tertiles of SAD(minimun-maximum, cm)	No. of events/No. of patients	Follow-up,No. of Person-Years	Event rate per100 Person-Years
**All-cause death**			
Tertile 1 (14.1–22.2)	11/139	434.5	2.5
Tertile 2 (22.3–26.9)	35/139	407.5	8.5
Tertile 3 (27.0–35.9)	51/140	350.4	14.5
**Cardiovascular death**			
Tertile 1 (14.1–22.2)	3/139	434.5	0.7
Tertile 2 (22.3–26.9)	20/139	407.5	4.9
Tertile 3 (27.0–35.9)	25/140	350.4	7.1

*Abbreviation:* SAD, sagittal abdominal diameter.

**Figure 1:**
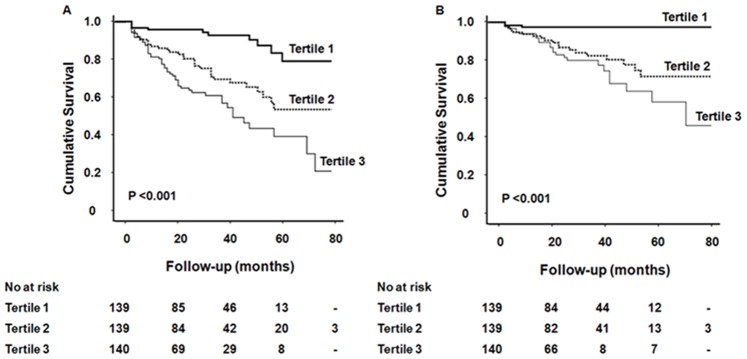
Kaplan-Meier analysis of (A) all-cause and (B) cardiovascular mortality according to the SAD tertiles. Patients with higher SAD tertiles showed significantly higher all-cause and cardiovascular mortality (both log-rank test, *P*<0.001). *Abbreviations*: SAD, sagittal abdominal diameter.

**Figure 2:**
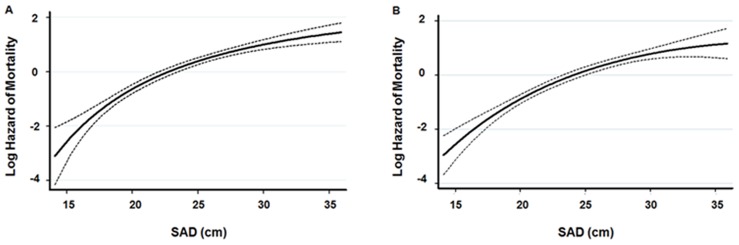
Multivariate fractional polynomial graphs for the association between SAD and (A) all-cause mortality and (B) cardiovascular mortality. Hazard ratios were calculated after adjustment for age, sex, diabetes mellitus, previous history of cardiovascular disease, smoking status, systolic blood pressure, the use of lipid-lowering therapy, BMI, and biochemical data (hemoglobin, albumin, Ca×P products, and log hs-CRP levels). Shaded areas indicate the 95% confidence limits. *Abbreviations*: SAD, sagittal abdominal diameter; BMI, body mass index; Ca, calcium; P, phosphorous; hs-CRP, high-sensitivity C-reactive protein.

**Figure 3:**
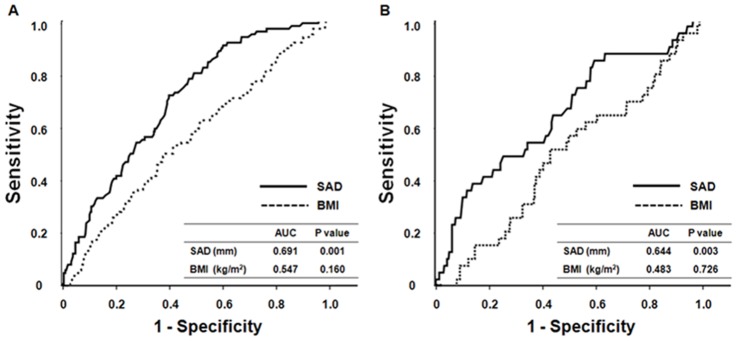
Receiver operating characteristic (ROC) curve for SAD and BMI to predict (A) all-cause and (B) cardiovascular mortality. SAD provided higher predictive accuracy for all-cause and cardiovascular mortality than BMI (both *P*<0.001). *Abbreviations*: SAD, sagittal abdominal diameter; BMI, body mass index; AUC, area under the ROC curve.

Kaplan-Meier analysis and Cox proportional hazard models were performed to determine the prognostic value of SAD on mortality. Both all-cause and cardiovascular mortality-free survival rates decreased significantly and gradually as SAD tertiles were increased (log-rank test, *P*<0.001) (Figure 1). In addition, univariate Cox proportional hazard analysis revealed that older age, the presence of diabetes and previous cardiovascular disease, the use of lipid-lowering drugs, higher serum hs-CRP levels, and lower serum albumin concentrations as well as higher SAD tertiles were significant risk factors for all-cause and cardiovascular mortality. In multivariate Cox analysis, the risks of all-cause and cardiovascular death were increased with increasing SAD tertiles. Relative to the lowest SAD tertile, the hazard ratios (HR) of all-cause and cardiovascular mortality in the highest tertile were 3.333 and 8.021, respectively (Table 4). When included as a continuous variable, SAD was a significant independent predictor of all-cause (per 1 cm increase, HR: 1.071, 95% CI: 1.005–1.141, *P* = 0.03) and cardiovascular mortality (per 1 cm increase, HR: 1.106, 95% CI: 1.007–1.214, *P* = 0.03). In addition, when SAD was evaluated in a fractional polynomial analysis, the risk of all-cause and cardiovascular death increased steadily with higher SAD values (Figure 2). Moreover, SAD provided higher predictive value for all-cause (AUC: 0.691 vs. 0.547, *P*<0.001) and cardiovascular mortality (AUC: 0.644 vs. 0.483, *P*<0.001) than BMI (Figure 3).

**Table 4:** Cox’s proportional hazard models of SAD for all-cause and cardiovascular mortality.

	Crude		Model 1^1^		Model 2^2^	
	HR (95% CI)	*P*	HR (95% CI)	*P*	HR (95% CI)	*P*
**All-cause mortality**						
SAD (per 1 cm)	1.143 (1.092–1.198)	<0.001	1.090 (1.022–1.163)	0.01	1.071 (1.005–1.141)	0.03
Tertile 1 (14.1–22.2)	1 (reference)		1 (reference)		1 (reference)	
Tertile 2 (22.3–26.9)	3.413 (1.733–6.721)	<0.001	1.975 (0.934–4.175)	0.07	2.366 (1.116–5.015)	0.03
Tertile 3 (27.0–35.9)	5.820 (3.028–11.188)	<0.001	3.538 (1.590–7.872)	0.01	3.333 (1.514–7.338)	0.01
**Cardiovascular mortality**						
SAD (per 1 cm)	1.160 (1.085–1.239)	<0.001	1.130 (1.030–1.240)	0.01	1.106 (1.007–1.214)	0.03
Tertile 1 (14.1–22.2)	1 (reference)		1 (reference)		1 (reference)	
Tertile 2 (22.3–26.9)	7.226 (2.146–24.323)	<0.001	5.535 (1.494–20.504)	0.01	5.955 (1.603–22.114)	0.01
Tertile 3 (27.0–35.9)	10.660 (3.221–35.385)	<0.001	10.690 (2.662–42.931)	0.01	8.021 (1.994–32.273)	0.01

*Abbreviations:* SAD, sagittal abdominal diameter; HR, hazard ratio; CI, confidence interval; BMI, body mass index.

1 Model 1: adjusted for age, sex, diabetes mellitus, previous history of cardiovascular disease, smoking, systolic blood pressure, and lipid-lowering agents, and BMI.

2 Model 2: Model 1+ biochemical data (hemoglobin, albumin, calcium×phosphorus products, and log high-sensitivity C-reactive protein levels).

### Prognostic Value of SAD for Mortality According to the Sex, Age, and BMI Subgroups

**Figure 4:**
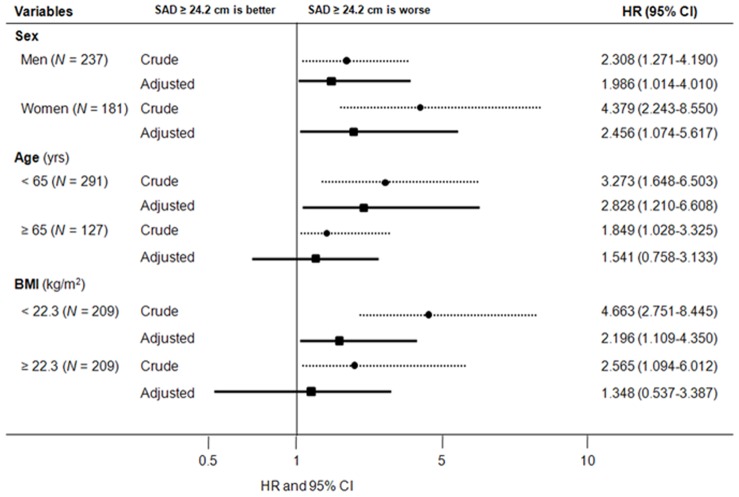
HRs of all-cause mortality for higher SAD (≥24.2 cm) in different subgroups of 418 patients. Higher SAD was significantly associated with increased risk of all-cause mortality in men, women, younger age (<65 years), and lower BMI groups (<22.3 kg/m^2^). Adjusted HRs were calculated after adjustment for age, sex, diabetes mellitus, previous history of cardiovascular disease, smoking, systolic blood pressure, the use of lipid-lowering therapy, BMI, and biochemical data (hemoglobin, albumin, Ca×P products, and log hs-CRP levels). *Abbreviations*: SAD, sagittal abdominal diameter; BMI, body mass index; Ca, calcium; P, phosphorous; hs-CRP, high-sensitivity C-reactive protein; HR, hazard ratio; CI, confidence interval.

The impact of SAD on mortality was further investigated according to sex, age, and BMI subgroups (Figure 4). In men, univariate Cox analysis revealed that older age, the presence of diabetes mellitus and previous cardiovascular disease, smoking, high BMI and serum hs-CRP levels, low serum albumin concentrations, and higher SAD (≥24.2 cm) were significant risk factors for all-cause mortality. In multivariate Cox analysis, higher SAD was a significant independent predictor of all-cause mortality (HR: 1.986, 95% CI: 1.014–4.010, *P* = 0.05). Subsequent subgroup analysis showed that the risk of all-cause mortality for the high SAD group was significantly higher in women (HR: 2.456, 95% CI: 1.074–5.617, *P* = 0.03). In addition, higher SAD was also significantly associated with all-cause mortality in the young (<65 years, HR: 2.828, 95% CI: 1.210–6.608, *P* = 0.01) and low BMI group (<22.3 kg/m^2^, HR: 2.196, 95% CI: 1.109–4.350, *P* = 0.03). Meanwhile, higher SAD was significantly associated with cardiovascular mortality only in women (HR: 6.017, 95% CI: 1.359–26.426, *P* = 0.02) and the young age group (HR: 2.269, 95% CI: 1.144–6.907, *P* = 0.04).

## Discussion

SAD is a simple and reliable anthropometric index representing visceral fat, which is closely associated with cardiovascular disease [18], [20], [21]. Since cardiovascular disease is the most common cause of death in ESRD patients [1], it is surmised that there may be an association between SAD and cardiovascular disease in these patients, but this hypothesis has never been explored. In the present study, we demonstrated for the first time that SAD on lateral abdominal X-ray at the start of PD was a significant independent predictor of all-cause and cardiovascular mortality in incident PD patients. In addition, the prognostic value of SAD was stronger than BMI in these patients.

The impact of obesity, which was defined by BMI, on all-cause and cardiovascular death has been widely investigated in the general population [3], [5]. However, several studies have revealed that BMI in overweight and mildly obese individuals was associated with lower mortality rates and fewer cardiovascular events, a phenomenon called the ‘obesity paradox’ [33], [34]. Even though the reason for obesity paradox has not been completely explained, several studies have suggested that it is attributed to overadjustment for confounders, lack of statistical power, and limitations of BMI to diagnose obesity [33], [35], [36]. In addition, it is well known that BMI cannot differentiate fat and lean mass and that BMI is affected by not only fat and muscle mass but also fluid status [33]. Based on these findings, the need for new surrogates to diagnose and provide better predictive value for all-cause and cardiovascular mortality has emerged.

Visceral or central obesity has been supposed to have a greater impact on cardiovascular events and mortality than total obesity, as demonstrated by numerous studies on healthy subjects and ESRD patients [11]–[17], [21]. Postorino et al [15] found that WC, but not BMI, was a significant independent predictor of all-cause and cardiovascular mortality in 537 ESRD patients. In prevalent PD patients, WHR also independently predicted mortality and the first cardiovascular event [16]. Consistent with most previous studies, we also showed that SAD, an index of central obesity, was a significant independent predictor of all-cause and cardiovascular mortality in incident PD patients, even after adjusting for BMI and that SAD provided higher predictive value for mortality than BMI. Moreover, our results revealed that SAD was significantly associated with serum hs-CRP concentrations. Furthermore, previous studies demonstrated that SAD had stronger correlations to cardio-metabolic risk factors than BMI [26]–[28], [37], [38]. Similarly, a study by Kalantar-Zadeh et al [39] found that the concentrations of inflammatory markers were comparable among the four different BMI groups of ESRD patients. SAD has also been known to be associated with insulin resistance [28], [37], [38], [40], [41]. Pouliot et al [41] showed that SAD was significantly associated with fasting glucose and postglucose insulin levels in 151 healthy subjects. In addition, SAD was also a significant predictor of insulins resistance in moderately obese men [37] and severely obese patients [28]. Moreover, SAD explained a greater portion of variation of insulin resistance and CRP levels in immigrant women from the Middle East and native Swedish women [38], and Chinese hypertensive patients [40]. Based on these findings, we speculated that a close relationship among visceral fat, cardio-metabolic risk, inflammation, insulin resistance, and SAD might contribute to the deleterious effect of SAD on mortality. On the other hand, higher SAD was significantly associated with all-cause mortality in the lower BMI group, but not the higher BMI group. Since the interaction between SAD and BMI was significantly stronger in the higher BMI group, we infer that the impact of SAD on mortality may be lessened in the higher BMI group.

SAD is the anteroposterior diameter of the abdomen, or ‘abdominal height’, and has been demonstrated to be closely related to visceral fat in previous studies [22]–[25]. Kvist et al [22] showed that SAD was a significant predictor of visceral fat volume determined by CT in 27 healthy subjects, indicating that SAD was useful in evaluating visceral obesity. The changes in SAD also correlated with the loss of visceral fat area on MRI in obese patients [23]. Furthermore, SAD had a stronger predictive value for identifying visceral fat than any other anthropometric indices [24], [25]. Similar to western subjects [22]–[24], SAD was revealed to have a stronger correlation with visceral fat area on CT compared to BMI, WC, and transverse abdominal diameter in 5,257 Korean subjects [25]. Since subcutaneous fat is displaced inferiorly by gravity in the supine position, SAD measured in the supine position could better represent visceral fat [22]. For these reasons, in the current study, we measured SAD by lateral abdominal X-ray taken in the supine position and used it as an index of central obesity. In addition, since the assessment was performed at fixed point of the L4-5 level on X-rays, we presumed that the intraobserver variability of SAD measurements by X-ray was less than using a caliper. In this study, the mean SAD was 25.2 cm in men, and 23.7 cm in women, which were higher than the Asian general population. The results of a study of 282 Japanese men and another study of 131 Chinese men revealed that the mean values of SAD were 18.7 and 19.5 cm, respectively [40], [42]. The mean SAD in women of our study was also higher than Chinese women (23.7 vs. 18.8 cm) [40]. Moreover, when patients were classified by sex and age, the mean SADs of the current study were higher than those of a study in 5,257 Korean general population (<50 years men, 23.7 vs. 22.7 cm; ≥50 years men, 23.3 vs. 22.6 cm; <50 years women, 21.0 vs. 18.5 cm; ≥50 years women, 25.0 vs. 20.0 cm) [25]. The reason why there is a disparity in SAD between the subjects of the present study and the Asian general population is not clear, but it is presumed that chronic inflammation status, more comorbid diseases such as diabetes, and low physical activity in incident ESRD patients may contribute to this difference.

In this study, even though total cholesterol levels were tended to have a negative correlation with SAD, total cholesterol concentrations were not different among the SAD tertiles. However, the proportion of patients taking statin was significantly higher in patients with higher SAD tertile. When we performed additional correlation tests according to the use of lipid-lowering therapy, SAD showed a significant positive association with total cholesterol levels in non-user of statin (statin user, *r* = −0.154, *P* = 0.31; non-user, *r = *0.082, *P = *0.011). Therefore, we surmised that these discrepant data might be attributed to lipid-lowering therapy. Meanwhile, the results of our study revealed that SAD was negatively associated with serum albumin concentrations. Hypoalbuminemia is a common complication in PD patients and the causes of hypoalbuminemia are complex such as aging, malnutrition, ultrafiltration failure, excess protein loss through the peritoneal membrane, and systemic inflammation [43], [44]. Since there was a significant positive association between hs-CRP levels and SAD, and hs-CRP levels were significantly higher in the highest SAD group, we suggested that enhanced inflammation in patients with higher SAD could be a possible reason of hypoalbuminemia in the higher SAD groups. However, these findings were the results of cross-sectional data analysis, the cause and effect relationship could not be determined. Furthermore, other data representing nutritional status, including measurement of daily food intake balance, normalized protein catabolic rate, or subjective global assessment were not available in the current study. In this regard, the association between SAD and nutritional status could not be completely elucidated in the present study.

The prognostic value of SAD has been investigated in only a few previous studies [29]–[31]. The Baltimore Longitudinal Study on Aging, which was a prospective study in 981 males at the National Institute on Aging in Baltimore, found that SAD was a strong predictor of all-cause and coronary heart disease mortality in young adults [29]. In 7,079 asymptomatic men in the Paris Prospective Study I, the risk of sudden death increased proportionally with increasing SAD quintile [30]. In addition, another cohort study on 101,765 subjects undergoing a health checkup identified that standing SAD was a strong predictor of coronary heart disease independently of BMI [31]. To date, however, the association of SAD with the clinical outcome has never been elucidated in ESRD patients. In this study, we show for the first time that SAD at PD initiation is a significant independent predictor of all-caue and cardiovascular mortality in ESRD patients. Moreover, while the majority of subjects were male in previous studies that demonstrated a good predictive value of SAD for the clinical outcome [29], [30], our study revealed that higher SAD was a significant independent predictor of mortality in both men and women. On the other hand, a significant association between higher SAD and mortality was observed in the younger age and lower BMI groups but not in the older age and higher BMI groups. The exact reason for this different impact of SAD is not clear, but it may be attributed to the possibility that SAD may exert more deleterious effect in patients with low risk of mortality compared to patients with high mortality risk.

The current study has several limitations. First, although previous studies found a significant association between SAD and visceral fat [22]–[25], CT and MRI were not performed to estimate visceral fat due to costs and radiation exposure. Second, other anthropometric indices, such as WHR and WC, were not evaluated, and the predictability of these indices for the clinical outcome was not compared with that of SAD. Third, patients on HD were not included. Since HD patients are generally followed up at private clinics but not at our institute, it was not easy to determine their final outcome. Finally, since SAD was determined only at the start of PD, the effect of changes in SAD during follow-up was not evaluated. Despite these limitations, we believe that this prospective observational study provides useful information on the effect of SAD on the clinical outcome in incident PD patients.

In conclusion, the present study demonstrates for the first time that SAD on lateral abdominal X-ray at PD commencement was a significant independent predictor for all-cause and cardiovascular mortality in incident PD patients. Considering the significant association of visceral fat with inflammation and cardiovascular risk, estimating visceral fat by SAD on lateral abdominal X-ray could be a useful tool to stratify mortality risk in these patients.

## References

[1] Foley RN, Parfrey PS, Sarnak MJ (1998) Clinical epidemiology of cardiovascular disease in chronic renal disease. Am J Kidney Dis 32: S112–119. 9820470 10.1053/ajkd.1998.v32.pm9820470

[2] Lamon-Fava S, Wilson PW, Schaefer EJ (1996) Impact of body mass index on coronary heart disease risk factors in men and women. The Framingham Offspring Study. Arterioscler Thromb Vasc Biol 16: 1509–1515. 8977456 10.1161/01.atv.16.12.1509

[3] Calle EE, Thun MJ, Petrelli JM, Rodriguez C, Heath CW Jr (1999) Body-mass index and mortality in a prospective cohort of U.S. adults. New Engl J Med 341: 1097–1105. 10511607 10.1056/NEJM199910073411501

[4] Wolk R, Berger P, Lennon RJ, Brilakis ES, Somers VK (2003) Body mass index: a risk factor for unstable angina and myocardial infarction in patients with angiographically confirmed coronary artery disease. Circulation 108: 2206–2211. 14557360 10.1161/01.CIR.0000095270.85646.E8

[5] Adams KF, Schatzkin A, Harris TB, Kipnis V, Mouw T, et al (2006) Overweight, obesity, and mortality in a large prospective cohort of persons 50 to 71 years old. New Engl J Med 355: 763–778. 16926275 10.1056/NEJMoa055643

[6] Leavey SF, McCullough K, Hecking E, Goodkin D, Port FK, et al (2001) Body mass index and mortality in ‘healthier’ as compared with ‘sicker’ haemodialysis patients: results from the Dialysis Outcomes and Practice Patterns Study (DOPPS). Nephrol Dial Transplant 16: 2386–2394. 11733631 10.1093/ndt/16.12.2386

[7] Kalantar-Zadeh K, Kopple JD, Kilpatrick RD, McAllister CJ, Shinaberger CS, et al (2005) Association of morbid obesity and weight change over time with cardiovascular survival in hemodialysis population. Am J Kidney Dis 46: 489–500. 16129211 10.1053/j.ajkd.2005.05.020

[8] McDonald SP, Collins JF, Johnson DW (2003) Obesity is associated with worse peritoneal dialysis outcomes in the Australia and New Zealand patient populations. J Am Soc Nephrol 14: 2894–2901. 14569099 10.1097/01.asn.0000091587.55159.5f

[9] Snyder JJ, Foley RN, Gilbertson DT, Vonesh EF, Collins AJ (2003) Body size and outcomes on peritoneal dialysis in the United States. Kidney Int 64: 1838–1844. 14531819 10.1046/j.1523-1755.2003.00287.x

[10] Abbott KC, Glanton CW, Trespalacios FC, Oliver DK, Ortiz MI, et al (2004) Body mass index, dialysis modality, and survival: analysis of the United States Renal Data System Dialysis Morbidity and Mortality Wave II Study. Kidney Int 65: 597–605. 14717930 10.1111/j.1523-1755.2004.00385.x

[11] Yusuf S, Hawken S, Ounpuu S, Bautista L, Franzosi MG, et al (2005) Obesity and the risk of myocardial infarction in 27,000 participants from 52 countries: a case-control study. Lancet 366: 1640–1649. 16271645 10.1016/S0140-6736(05)67663-5

[12] de Koning L, Merchant AT, Pogue J, Anand SS (2007) Waist circumference and waist-to-hip ratio as predictors of cardiovascular events: meta-regression analysis of prospective studies. Eur Heart J 28: 850–856. 17403720 10.1093/eurheartj/ehm026

[13] Elsayed EF, Tighiouart H, Weiner DE, Griffith J, Salem D, et al (2008) Waist-to-hip ratio and body mass index as risk factors for cardiovascular events in CKD. Am J Kidney Dis 52: 49–57. 18514990 10.1053/j.ajkd.2008.04.002 PMC2693892

[14] Zeller M, Steg PG, Ravisy J, Lorgis L, Laurent Y, et al (2008) Relation between body mass index, waist circumference, and death after acute myocardial infarction. Circulation 118: 482–490. 18625893 10.1161/CIRCULATIONAHA.107.753483

[15] Postorino M, Marino C, Tripepi G, Zoccali C (2009) Abdominal obesity and all-cause and cardiovascular mortality in end-stage renal disease. J Am Coll Cardiol 53: 1265–1272. 19358939 10.1016/j.jacc.2008.12.040

[16] Su WS, Clase CM, Brimble KS, Margetts PJ, Wilkieson TJ, et al (2010) Waist-to-Hip Ratio, Cardiovascular Outcomes, and Death in Peritoneal Dialysis Patients. Int J Nephrol 5: 831243. 10.4061/2010/831243 PMC3003982 21188241

[17] Coutinho T, Goel K, Correa de Sa D, Kragelund C, Kanaya AM, et al (2011) Central obesity and survival in subjects with coronary artery disease: a systematic review of the literature and collaborative analysis with individual subject data. J Am Coll Cardiol 57: 1877–1886. 21545944 10.1016/j.jacc.2010.11.058

[18] Ribeiro-Filho FF, Faria AN, Kohlmann O Jr, Ajzen S, Ribeiro AB, et al (2001) Ultrasonography for the evaluation of visceral fat and cardiovascular risk. Hypertension 38: 713–717. 11566963 10.1161/01.hyp.38.3.713

[19] Zoccali C, Mallamaci F, Tripepi G (2004) Inflammatory proteins as predictors of cardiovascular disease in patients with end-stage renal disease. Nephrol Dial Transplant 19 Suppl 5V67–72. 15284363 10.1093/ndt/gfh1059

[20] Kim SK, Park SW, Kim SH, Cha BS, Lee HC, et al (2009) Visceral fat amount is associated with carotid atherosclerosis even in type 2 diabetic men with a normal waist circumference. Int J Obes (Lond) 33: 131–135. 18982015 10.1038/ijo.2008.222

[21] Lee MJ, Shin DH, Kim SJ, Oh HJ, Yoo DE, et al (2012) Visceral fat thickness is associated with carotid atherosclerosis in peritoneal dialysis patients. Obesity (Silver Spring) 20: 1301–1307. 21818151 10.1038/oby.2011.245

[22] Kvist H, Chowdhury B, Grangard U, Tylen U, Sjostrom L (1988) Total and visceral adipose-tissue volumes derived from measurements with computed tomography in adult men and women: predictive equations. Am J Clin Nutr 48: 1351–1361. 3202084 10.1093/ajcn/48.6.1351

[23] van der Kooy K, Leenen R, Seidell JC, Deurenberg P, Visser M (1993) Abdominal diameters as indicators of visceral fat: comparison between magnetic resonance imaging and anthropometry. Br J Nutr 70: 47–58. 8399118 10.1079/bjn19930104

[24] Clasey JL, Bouchard C, Teates CD, Riblett JE, Thorner MO, et al (1999) The use of anthropometric and dual-energy X-ray absorptiometry (DXA) measures to estimate total abdominal and abdominal visceral fat in men and women. Obes Res 7: 256–264. 10348496 10.1002/j.1550-8528.1999.tb00404.x

[25] Yim JY, Kim D, Lim SH, Park MJ, Choi SH, et al (2010) Sagittal abdominal diameter is a strong anthropometric measure of visceral adipose tissue in the Asian general population. Diabetes Care 33: 2665–2670. 20843976 10.2337/dc10-0606 PMC2992209

[26] Richelsen B, Pedersen SB (1995) Associations between different anthropometric measurements of fatness and metabolic risk parameters in non-obese, healthy, middle-aged men. Int J Obes Relat Metab Disord 19: 169–174. 7780492

[27] Gustat J, Elkasabany A, Srinivasan S, Berenson GS (2000) Relation of abdominal height to cardiovascular risk factors in young adults: the Bogalusa heart study. Am J Epidemiol 151: 885–891. 10791561 10.1093/oxfordjournals.aje.a010292

[28] Guzzaloni G, Minocci A, Marzullo P, Liuzzi A (2009) Sagittal abdominal diameter is more predictive of cardiovascular risk than abdominal fat compartments in severe obesity. Int J Obes (Lond) 33: 233–238. 19139755 10.1038/ijo.2008.271

[29] Seidell JC, Andres R, Sorkin JD, Muller DC (1994) The sagittal waist diameter and mortality in men: the Baltimore Longitudinal Study on Aging. Int J Obes Relat Metab Disord 18: 61–67. 8130817

[30] Empana JP, Ducimetiere P, Charles MA, Jouven X (2004) Sagittal abdominal diameter and risk of sudden death in asymptomatic middle-aged men: the Paris Prospective Study I. Circulation. 110: 2781–2785. 10.1161/01.CIR.0000146395.64065.BA 15492315

[31] Iribarren C, Darbinian JA, Lo JC, Fireman BH, Go AS (2006) Value of the sagittal abdominal diameter in coronary heart disease risk assessment: cohort study in a large, multiethnic population. Am J Epidemiol 164: 1150–1159. 17041127 10.1093/aje/kwj341

[32] Lang RM, Bierig M, Devereux RB, Flachskampf FA, Foster E, et al (2005) Recommendations for chamber quantification: a report from the American Society of Echocardiography’s Guidelines and Standards Committee and the Chamber Quantification Writing Group, developed in conjunction with the European Association of Echocardiography, a branch of the European Society of Cardiology. J Am Soc Echocardiogr 18: 1440–1463. 16376782 10.1016/j.echo.2005.10.005

[33] Romero-Corral A, Montori VM, Somers VK, Korinek J, Thomas RJ, et al (2006) Association of bodyweight with total mortality and with cardiovascular events in coronary artery disease: a systematic review of cohort studies. Lancet 368: 666–678. 16920472 10.1016/S0140-6736(06)69251-9

[34] Oreopoulos A, McAlister FA, Kalantar-Zadeh K, Padwal R, Ezekowitz JA, et al (2009) The relationship between body mass index, treatment, and mortality in patients with established coronary artery disease: a report from APPROACH. Eur Heart J 30: 2584–2592. 19617221 10.1093/eurheartj/ehp288 PMC2771146

[35] Greenberg JA (2006) Correcting biases in estimates of mortality attributable to obesity. Obesity (Silver Spring) 14: 2071–2079. 17135625 10.1038/oby.2006.242

[36] Romero-Corral A, Somers VK, Sierra-Johnson J, Thomas RJ, Collazo-Clavell ML, et al (2008) Accuracy of body mass index in diagnosing obesity in the adult general population. Int J Obes (Lond) 32: 959–966. 18283284 10.1038/ijo.2008.11 PMC2877506

[37] Risérus U, Arnlöv J, Brismar K, Zethelius B, Berglund L, et al (2004) Sagittal abdominal diameter is a strong anthropometric marker of insulin resistance and hyperproinsulinemia in obese men. Diabetes care 27: 2041–2046. 15277437 10.2337/diacare.27.8.2041

[38] Petersson H, Daryani A, Risérus U (2007) Sagittal abdominal diameter as a marker of inflammation and insulin resistance among immigrant women from the Middle East and native Swedish women: a cross-sectional study. Cardiovasc Diabetol 6: 10. 17391519 10.1186/1475-2840-6-10 PMC1847804

[39] Kalantar-Zadeh K, Kuwae N, Wu DY, Shantouf RS, Fouque D, et al (2006) Associations of body fat and its changes over time with quality of life and prospective mortality in hemodialysis patients. Am J Clin Nutr 83: 202–210. 16469976 10.1093/ajcn/83.2.202

[40] Hwu CM, Hsiao CF, Sheu WH, Pei D, Tai TY, et al (2003) Sagittal abdominal diameter is associated with insulin sensitivity in Chinese hypertensive patients and their siblings. J Hum Hypertens 17: 193–198. 12624610 10.1038/sj.jhh.1001532

[41] Pouliot MC, Despres JP, Lemieux S, Moorjani S, Bouchard C, et al (1994) Waist circumference and abdominal sagittal diameter: best simple anthropometric indexes of abdominal visceral adipose tissue accumulation and related cardiovascular risk in men and women. Am J Cardiol 73: 460–468. 8141087 10.1016/0002-9149(94)90676-9

[42] Nakata K, Choo J, Hopson MJ, Ueshima H, Curb JD, et al (2010) Stronger associations of sagittal abdominal diameter with atherogenic lipoprotein subfractions than waist circumference in middle-aged US white and Japanese men. Metabolism 59: 1742–1751. 20580038 10.1016/j.metabol.2010.04.019 PMC2978280

[43] Cueto-Manzano AM, Espinosa A, Hernández A, Correa-Rotter R (1997) Peritoneal transport kinetics correlate with serum albumin but not with the overall nutritional status in CAPD patients. Am J Kidney Dis 30: 229–236. 9261034 10.1016/s0272-6386(97)90057-3

[44] Shioya M, Yoshida T, Kasai K, Furuya R, Kato A, et al (2013) Inflammatory factors for hypoalbuminemia in Japanese peritoneal dialysis patients. Nephrology (Carlton) 18: 539–544. 23718260 10.1111/nep.12106

